# Anal Cancer Prevention Perspectives Among Foreign-Born Latino HIV-Infected Gay and Bisexual Men

**DOI:** 10.1177/1073274818780368

**Published:** 2018-06-21

**Authors:** Alexis M. Koskan, Madeline Fernandez-Pineda

**Affiliations:** 1 Arizona State University, College of Health Solutions, School of Nutrition and Health Promotion, Phoenix, AZ, USA; 2 School of Nursing and Health Studies, University of Miami, Coral Gables, FL, USA

**Keywords:** human papillomavirus, anal cancer, high-grade squamous intraepithelial lesions, anal intraepithelial neoplasia, gay and bisexual men, cancer prevention

## Abstract

This study explores understanding of primary and secondary prevention of anal cancer among human immunodeficiency virus (HIV)-infected foreign-born Latino gay and bisexual men (GBM). Between August 2015 and December 2016, researchers conducted 33 in-depth, semi-structured interviews with HIV-infected foreign-born Latino GBM. Interview questions sought to determine participants’ knowledge and perceived barriers and facilitators to primary and secondary prevention of anal cancer. Researchers analyzed interview transcripts using a qualitative content analysis approach. For primary prevention, men reported a lack of knowledge about the human papillomavirus (HPV) vaccine. However, for secondary prevention, roughly 60% of participants had previously screened for anal dysplasia via anal Papanicolaou (Pap) smear. However, participants reported willingness to screen, and provider recommendation was the most common screening facilitator. Men reported stigma related to their HIV status, sexual orientation, and anal Pap smear procedures as anal cancer screening barriers. Participants reported willingness to use a self-screening anal Pap smear test if it was commercially available. Health providers continue to be the leading source of health information. Therefore, provider recommendation for HPV vaccination and anal cancer screening among age-eligible foreign-born Latino HIV-infected GBM is critical. More work is needed to destigmatize HIV and sexual orientation to influence positive health behaviors among this population. Future intervention research could test the effects of provider-led interventions and also media campaigns aimed at influencing HPV vaccine uptake and anal cancer screening among this population.

## Introduction

Rates of anal cancer, a relatively rare disease, which account for 0.5% of all new cases with cancer, have been steadily rising by 2.2% each year.^1^ Between 2016 and 2017 in the United States alone, anal cancer incidence increased from 8080 to 8200 new cases, and anal cancer mortality increased from 1080 to 1100 deaths.^1^ The majority (91%) of anal cancers are squamous cell carcinomas caused by persistent infection of the anal canal with high-risk strains (types 16 and 18) of human papillomavirus (HPV), the most common sexually transmitted infection worldwide.^2^ Persistent infection of the anal canal, particularly with HPV 16, may lead to the development of abnormal cell growth, known as anal dysplasia. Most individuals exposed to HPV clear the infection without ever showing signs or symptoms. However, human immunodeficiency virus (HIV)-infected and other immunosuppressed populations experience an increased risk of persistent HPV infection.^3^ Over time, dysplasia can progress to high-grade squamous intraepithelial lesions (HSILs).^4^ Researchers and health-care practitioners believe that, similar to cervical HSIL and cervical cancer, untreated anal HSIL is the cause of anal cancer.^5^ The 2 greatest risk factors for developing anal cancer are the presence of anal HSIL^6^ and severe immunosuppression (CD4 count <200 cells/mm^3^).^7-10^ Other risk factors include age (being older than age 25),^11^ smoking (linked to prolonged infection with HPV oncogenic strains),^12^ infection with multiple high-risk HPV strains,^13^ increasing number of current sexual partners,^14^ history of developing anal warts,^15^ higher HIV viral loads,^15^ and reported regular receptive anal sex.^15^

It is believed that HPV-related anal cancer can be prevented via primary (HPV vaccination) and secondary (anal dysplasia and HSIL screenings) prevention. For primary prevention, two commercially available HPV vaccines (a 9-valent vaccine and a 4-valent vaccine) offer the potential for immunity against HPV 16 and 18 (oncogenic strains) as well as HPV 6 and 11 (nononcogenic strains that cause the majority of genital warts).^16,17^ To be most effective in preventing disease and saving money, all individuals should receive the vaccine prior to sexual debut.^18^ The vaccine is also safe and recommended for HIV-infected individuals between the ages of 9 and 26 years.^19^

For secondary anal cancer prevention, the anal Papanicolaou (Pap) smear is recommended as a first screening test to detect anal dysplasia among individuals asymptomatic for anal cancer and particularly for HIV-infected individuals.^20^ While high-resolution anoscopy (HRA) is a more sensitive and specific screening for detecting HSIL, numerous barriers (eg, lack of provider training to perform the test and lack of HRA equipment) prohibit using HRA as the initial screening test.^21,22^ Although there are no national guidelines for anal dysplasia/anal cancer screening, researchers have found that screening HIV-infected populations for anal dysplasia every 2 to 3 years via anal Pap smear is a cost-effective, life-saving approach to detect (and potentially treat) early signs of anal cancer.^23^

HIV-infected men who have sex with men (MSM) are the population most at risk of anal cancer, given their immunosuppression, anal sex receptivity, and exposure to multiple high-risk HPV strains (including HPV 16).^24^ Past research identified barriers and facilitators to screening for anal cancer among HIV-infected gay and bisexual men (GBM) and MSM (predominantly non-Hispanic White) populations.^25,26^ Barriers included lack of awareness to screen, fear, and stigma related to receiving the anal Pap smear and cost of screening.

Little is known about HIV-infected foreign-born Latino GBM’s risk of anal cancer. This population has previously reported inconsistent use of condoms during anal sex, which increases the risk of sexually transmitted infections, including HPV.^27^ Among HIV-infected populations, foreign-born Latinos (including MSM)^28^ are the population least likely to be engaged and sustained in HIV primary care.^29^ Lack of sustained HIV primary care is associated with lower CD4 counts in HIV-infected patients’.^30^ Interruption of HIV primary care also places patients at a greater risk of other comorbidities (such as anal dysplasia), because patients are not being screened and treated for disease precursors.^31^ Although one literature review explored US Latino (including Puerto Rican) men’s HPV-related comorbidities,^32^ no studies, to our knowledge, report foreign-born Latino HIV-infected GBM’s perceptions about anal cancer prevention. Therefore, the objective of this study was to explore the perspectives, barriers, and facilitators related to both primary (HPV vaccine uptake) and secondary (anal cancer screening) prevention among foreign-born (not including Puerto Rican populations) Latino HIV-infected GBM. It is critical to understand the social determinants of these disparities among Latino immigrant populations in order to identify how to prevent and control anal cancer.

## Methods

Upon receiving institutional review board approval from the University of Miami (IRB00000260) to conduct the study, the research team began recruiting a convenience sample of Latino HIV-infected GBM. They circulated Spanish-language study fliers to community-based organizations that provide outreach services to Latino GBM. Staff from the community-based organizations posted fliers at their organizations. The fliers provided information about the topic of interest (cancer prevention), inclusion criteria (HIV-infected, self-identify as a gay or bisexual man, 18 years of age or older, fluent in Spanish [a previous study was conducted in English],^33^ and resides in Miami-Dade County), and a Google number that rang directly to the bilingual research assistant’s cellphone. After potential participants called the study number, the bilingual research assistant screened them for eligibility and asked them to recommend a date, time, and location for the interview.

This study utilized qualitative in-depth interviews to explore HIV-infected Latino GBM’s perceptions related to the HPV vaccine and anal cancer screening. The first author created an English-language in-depth interview guide. A professional transcription and translation company translated and back-translated the interview guide to ensure its linguistic accuracy. The bilingual research team members conducted in-depth interviews between August 2015 and December 2016. They met individually with each participant at a time and location of his choice. After obtaining written informed consent, the researcher verbally administered a demographic questionnaire before conducting the in-depth interview. See Table 1 for an English-language version of the interview guide. After the interview, the research team provided participants $60 [USD] as remuneration for their time and shared experiences.

**Table 1.** In-Depth Interview Guide Questions.

What have you heard about the virus HPV? How and where did you find out this information? How do you think you could be affected by HPV? What have you heard about the HPV vaccine? Where did you hear about it? Have you received the HPV vaccine? What do you think influences people to get the HPV vaccine? What keeps them from getting the vaccine? Is there anything else? What other information about the HPV vaccine would you like to share? What do you know about cancers related to HPV? What have you heard about anal cancer? Have you ever been asked to screen for anal cancer? If so, by whom? What did that person tell you? What would influence you to screen for anal cancer? What would stop you from screening for anal cancer? What other information about anal cancer would you like to share?

Abbreviation: HPV, human papillomavirus.

The first author sent the interview audio files to a local transcription company, which translated and transcribed the Spanish-language in-depth interview data. The research team conducted interviews until they reached data saturation, when no new information or themes had emerged from the data. The research team only analyzed interview transcripts of foreign-born Latino MSM. The first author created a preliminary coding guide using a priori themes (from the in-depth interview guide) that she and the bilingual research assistant expanded after conducting interactive rounds of coding together to achieve coder agreement. They conducted qualitative content analysis using an “intuitive” approach by reviewing all interview data and selecting the aspects most relevant to the research questions.^34^ They coded one transcript together using the coding guide, discussed coding discrepancies, independently coded three additional transcripts, and met in person to compare codes and remedy discrepancies. Upon reaching a consensus and expanding the coding guide, they split the remaining transcripts, hand-coded them, and entered the transcripts into ATLAS.ti software to manually assign codes and organize the qualitative themes that emerged from the data.

## Results

Researchers interviewed 33 foreign-born Latino HIV-infected GBM. See Table 2 for participant demographic information. Roughly half of the men had heard about HPV. Only one participant had received a single dose of the HPV. As for anal dysplasia screening, 20 men previously screened via anal Pap smear. Two men tested positive for HSIL, one of whom currently receives anal tissue biopsies once every three months. The other was diagnosed and treated for anal cancer via surgery and medicated ointments. The results below provide a more in-depth description of the study’s findings. See Table 3 for illustrative quotes for each theme.

**Table 2.** Participant Demographics.

Participants (N = 33)	
Age in years	Range = 22-68
	Mean = 44
	21-30: 9 (27.3%)
	31-40: 4 (12.1%)
	40-50: 9 (27.3%)
	51-60: 7 (21.2%)
	61+: 4 (12.1%)
Country of origin	Chile: 1 (3.0%)
	Colombia: 4 (12.1%)
	Cuba: 17 (51.5%)
	Dominican Republic: 1 (3.0%)
	Honduras: 2 (6.1%)
	Mexico: 4 (12.1%)
	Venezuela: 4 (12.1%)
Years lived in the United States	Range (6-49 years); Mean = 18.4 years
Education	Less than high school: 4 (12.1%)
	High school/GED: 7 (21.2%)
	Some college: 13 (39.4%)
	Bachelor’s degree: 7 (21.2%)
	Graduate degree: 2 (6.1%)
Employment	Employed for wages: 16 (48.5%)
	Self-employed: 3 (9.1%)
	Unemployed/unable to work: 10 (30.3%)
	Retired: 4 (12.1%)
Annual income	Less than USD20 000: 18 (54.5%)
	USD20 000-USD39 999: 13 (39.4%)
	USD40 000-USD70 000: 2 (6.1%)

Abbreviations: GED, general education diploma; HPV, human papillomavirus.

**Table 3.** Barriers and Facilitators to Anal Cancer Prevention.

Theme	Subtheme	Supporting Quote
HPV-related Knowledge	Knowledge about HPV	Personally, I’m 30 years old and I had never heard about it until my friend told me and that’s when I did some research. It would be great if people were informed. (Participant 4, Colombia, Age 30)
	Does not affect men	Well, I had heard about it when I was in high school. I didn’t know how it was with gay men, particularly in terms of how common it was, and now I know it’s really common and that it’s—there’s really no vaccine for it. I mean, there is for women but it’s until a certain age, from what I understand, and, yeah. I mean, you’re basically treated (Participant 21, Colombia, Age 25)
	Anal warts	Well, I have heard that it develops over the years and your body also produces it. I had a boyfriend once, he was positive and three years later he got some warts. The doctor told him he had a problem, I freaked out because I said—not only does he have HIV but he also has this. I thought it was contagious. But now I actually think that it’s a skin condition that is more or less normal. (Participant 8, Venezuela, Age 43)
Facilitators to HPV vaccine	Provider recommendation	Out here, the primary care is always speaking to them, and they [the Hispanic patients] really listen. Even though you think they’re not listening, they’re listening. I’m a peer educator, and I ask patients to consult with the doctor. After health care visits they tell me “Oh, I’m scared because the doctor just told me about such-and-such. (Participant 20, Honduras, Age 26)
Barriers to HPV vaccine	Lack of knowledge	People don’t have the least idea [about anal cancer], just like me. (Participant 2, Mexico, Age 44)
	Health insurance	Sometimes it’s the health insurance and also because they don’t go to the doctor. Because I know a lot of people that want to come and get a checkup but they can’t because they don’t have insurance, others are busy with work. But more than anything because they don’t have insurance. (Participant 10, Chile, Age 68)
	Side effects	That they tell you that it has effects—that it’s going to cause fevers or that you’re going to feel bad (Participant 20, Honduras, Age 26)
Barriers to screening for anal dysplasia	Stigma associated with sexual orientation	I’ve lived here 30 some years and it’s so closeted. I believe the culture, the Latin culture just has it so—they want to be open and out, there’s so much on the down low in this county. I dated a man for ten years who was married with kids. (Participant 31, Dominican Republic, Age 53)
	How sexual orientation is defined	There is a taboo about men having sex with men. A lot of men do not consider themselves as homosexuals, but they have sex with other men. (Participant 9, Cuba, Age 46)
	Intersectionality of sexual orientation and HIV stigma	I am from Venezuela is not much approved of, it is not accepted. This is the reason that the homosexuals begin have the habit of hiding. That world—it’s an underground world. Everything is hidden. As a homosexual, you are not accepted and not respected. Now, imagine how it would be to have HIV. (Participant 11, Venezuela, Age 45)
	Embarrassment of testing procedure	Mostly embarrassment. Yeah. You don’t want anybody playing with your butt. Right? I mean, let’s be honest. (Participant 20, Honduras, Age 26)
		I was embarrassed the first time. I was forty-three years old. The doctor put his finger inside me and I pushed it out. I was like, “No, no”, but after that I was like, “I have to do this because otherwise I will never know if I am well”. (Participant 1, Cuba, Age 56)
	Perceptions of how the test will impact masculinity	I think we believe that performing this test will make you less of a man. (Participant 4, Colombia, Age 30)
	Fear of anal dysplasia testing results	I would be scared to have a positive result. I wouldn’t know how to react about that but not as bad as with HIV. (Participant 5, Cuba, Age 59)
Self-screening for anal dysplasia	Prefers self-screening	It would be easier because there’s no fear of going to a doctor and explain everything for example nowadays there’s a test called Orasure test or Oraquick, and you can find it in pharmacies and it’s easier for people to take the test and then to go to the doctor. (Participant 5, Cuba, Age 59)
	Prefers health care providers conduct screening	I’d prefer the clinic because in case I don’t consider something and I infect the exam and the results would be wrong. I’d prefer this is done by the right person and in the right place. That’s something important because they’re detecting a cancer, and for people like us with a weak immune system will be better to go to the right place. (Participant 27, Cuba, Age 37)

Abbreviations: HIV, human immunodeficiency virus; HPV, human papillomavirus.

### Knowledge/Awareness about HPV and the HPV Vaccine

Fifteen participants described that HPV is transmitted sexually. Five of these men had never heard of the HPV vaccine. Six participants who knew that HPV was sexually transmitted described the virus as affecting both men and women, whereas 4 believed it was a women’s health issue only (see Table 3 to read quotes about HPV-related knowledge). Five knew that certain strains cause genital warts and anal cancer. Three other participants confused HPV with HIV. The remaining participants reported that they did not know anything about HPV (including that it was a sexually transmitted infection), the existence of the HPV vaccine, or about HPV-related cancers that also affect men (see Table 3). Among those who were less aware about HPV, some guessed that the best way to prevent the virus is through practicing healthy lifestyle behaviors such as eating a balanced diet, taking medications as needed, and engaging in regular physical activity. Participants were interested in receiving more information about these health-related topics, particularly information about how they can prevent cancer.

Fifteen participants had heard of the HPV vaccine, one of whom (out of the 9 men who had been age-eligible to receive the vaccine) had received a single dose of the HPV vaccine. When asked where they first heard of the vaccine, 6 participants reported they received this information from their health-care providers, 4 from television public service announcements, and 2 from watching the news. The remaining participants reported hearing about the vaccine from reading a newspaper article, attending a health-related conference, and attending an HIV support group (single responses). The greatest barrier to receiving the vaccine was lack of knowledge about this resource (n = 10). (See Table 3 to read quotes that describe patient-related barriers to receiving the HPV vaccine.) Other barriers included health insurance coverage (n = 5), fear of side effects (n = 4), and current low immune functioning (n = 2), which they believed could lead to further health complications if they received the vaccine. However, once the men learned about the availability of a vaccine that could reduce their chances of getting genital warts and certain cancers, they were interested in learning more about this resource.

### Anal Cancer Screening

Most participants had heard (n = 21) about anal cancer screening, 20 of whom reported having been screened for the cancer via anal Pap smear. These men described how provider recommendations influenced their decision to be screened. The 1 person who reported knowing about the anal Pap smear test but who had not actually been screened for anal dysplasia described how his health-care provider discouraged him from getting screened for anal dysplasia, telling him that he was too young to be screened.

### Psychosocial Barriers to Anal Cancer Screening

Psychosocial barriers to anal cancer screening included various forms of stigma, embarrassment, or discomfort related to anal dysplasia screening tests and fear of test results (see Table 3). Less frequently reported barriers included fear of seeking health care if undocumented (due to fear of deportation), being from a culture that promotes treatment as opposed to disease prevention, and drug use.

Multiple forms of stigma prevented men from seeking HIV primary care and anal cancer screening. Specifically, participants reported stigma surrounding the following conditions as barriers to receiving sustained HIV primary care: (1) sexual orientation (being a gay or bisexual man), (2) HIV infection, and (3) the combined stigma of being HIV-infected and identifying as gay/bisexual. Two men reported that self-perception or self-classification of being gay or bisexual influenced health-seeking behaviors, particularly if their providers told that being gay or bisexual increased their risk of negative health outcomes. They described how not all MSM regard themselves as gay or bisexual, and they also do not tell their providers that they are having sex with other men. In turn, providers may not promote the anal Pap smear to these patients. Four participants described the stigma against HIV as a reason why men did not seek HIV primary care. One participant described how the intersectionality of being a gay man who was HIV-positive increased his perceived stigma and fear of engaging in health-care practices, including screening for cancers.

Five participants described embarrassment-related screening procedures (specifically the anal Pap smear and digital rectal examinations) as a reason why Latino men would choose not to test for precursors to anal cancer. These procedures both involve touching the anal canal. According to participants, this can embarrass men. This type of procedure may also violate cultural norms of masculinity, also known as *machismo*. In addition, 4 men reported fear of receiving a cancer diagnosis as motivation to avoid anal cancer screening.

### Anal Dysplasia Self-Screening

Many participants (n = 20) were in favor of testing for anal cancer from home (via self-sampling for anal dysplasia) if it was a viable, commercially- available testing option (see Table 3). They noted that this was a more convenient form of screening because they could collect the sample themselves without the embarrassment of having a health care provider collect the sample, and they could do so within the comfort of their own homes, which lowered their fear of being stigmatized. However, not all participants were enthusiastic about self-sampling for anal cytology. Six participants preferred for a clinician to conduct the test, citing that a health care provider would most likely perform this test more accurately. Other participants were indifferent (n = 7) about this method of screening.

### Education Preferences

Participants recommended various methods to disseminate health information about HPV, the HPV vaccine, and anal cancer. They most commonly recommended (n = 21) that health care providers inform patients about these health issues and provide the option to receive the vaccine and screen for anal cancer. Other common responses included using media such as Spanish-language online resources (n = 10), television public service announcements (n = 8), and Spanish-language radio shows (n = 2) to reach the local community with information about HPV. Eight participants also recommended various forms of print media (eg, posters, brochures, or informational fliers) as health education resources.

## Discussion

To our knowledge, this is the first study to focus exclusively on foreign-born Latino HIV-infected GBM’s perceptions related to HPV, the HPV vaccine, and anal cancer screening. By extending cultural considerations for immigrant Latino GBM, our study’s findings add to the existing literature on various HIV-positive communities’ perceptions related to the prevention and control of anal cancer.^27,39^ Similar to those studies, this research found high levels of HPV vaccine acceptability. The only causes for vaccine hesitancy among foreign-born Latino HIV-infected GBM were vaccine costs and fears of potential health complications due to their HIV status. No additional negative side effects from the vaccine (which does not consist of the live virus) have been reported among HIV-infected populations when compared to the general population.^41^ Human immunodeficiency virus-infected adults often test negative for oncogenic strains of HPV. Over time, however, they increase their risk of acquiring cancerous HPV strains if they increase their number of sexual partners.^15^ Further, past studies have reported that completing the HPV vaccine can reduce the risk of HSIL recurrence among MSM previously treated for these precancerous lesions.^42^ Given its safety for immunosuppressed populations, the HPV vaccine should be promoted, particularly by HIV primary care clinics, to all age eligible (up to age 26 years old). HIV-infected MSM (including those who identify as GBM). Completing this vaccine series will help reduce their risk of developing various HPV-related cancers and noncancerous genital warts. Social marketing campaigns could conduct community-engaged formative work with HIV-positive Latino advocates (including foreign-born GBM) to determine the best methods for disseminating this health information in culturally and linguistically appropriate methods and language.^35^ Also, future studies should test the efficacy and cost-effectiveness of offering the HPV vaccine to all HIV-infected adults (including individuals over age 26) to reduce HPV-related cancer risk and recurrence.

In this sample, more than half of the participants had previously been screened for anal dysplasia via anal Pap smear, a higher rate than what was previously reported in another study with English-speaking populations.^33^ Foreign-born Latino HIV-infected GBM noted that health-care provider recommendation alone was the driving factor for anal Pap smear completion. Therefore, it is critical that HIV primary care providers offer culturally sensitive anal dysplasia screening information for this population. It is important to note that, as described by research participants, not all foreign-born Latino MSM identify as being gay or bisexual. Past research studies have also confirmed that Latino men who have sex with other men do not necessarily self-identify as gay or bisexual.^36,37^ Therefore, social marketing campaigns that aim to link foreign-born Latino GBM to HIV-related health care and to anal Pap smears may be more effective if they market the information to MSM, without focusing on the language related to sexual orientation.^36^

Participants described how the anal cytology testing performed by a health-care provider could violate their sense of masculinity. Past studies have found similar barriers to colorectal cancer screening, predominantly among Latino and African American populations.^38,39^ Gwede and colleagues recommend promoting a home-based self-sampling cancer-screening tool as an early detection tool for colorectal cancer to overcome embarrassment related to screening procedures.^39^ Similarly, using an anal cytology self-sampling test that may be collected at home could potentially increase anal cancer screenings. When asked about this testing procedure’s acceptability, most participants believed that a home self-sampling test could reduce barriers to screening. Such tests are not currently commercially available. However, similar to colorectal cancer screening, home self-testing kits may increase anal dysplasia screenings if made available.

Participants discussed the psychosocial barriers that could lead to a lack of screening and treatment for both HIV- and HPV-related comorbidities among Latino GBM. Although not specifically asked, men described various forms of stigma that are associated with sexual orientation, HIV status, and the intersection of sexuality and HIV status as barriers to care. Past research has also described the intersectionality of stigma related to HIV status, sexual orientation, and race as barriers to retention in HIV care among African American and Latino MSM.^40,41^ Together, these various forms of stigma lead to social isolation, depression, and lack of retention in HIV primary care,^42^ and they are correlated with increased sexual risk behaviors.^43^ Culturally tailored community-engaged interventions aimed at reducing homophobia and these various forms of stigma may increase Latino HIV-infected GBM’s retention in HIV primary care and, in turn, increase cancer-screening behaviors.^44^

There are potential limitations to this study. First, researchers asked foreign-born Latino GBM about HPV vaccine perspectives. At the time, only 9 participants had been age eligible (younger than age 26) to receive the vaccine since vaccine was first promoted for men (in October 2009). Therefore, it is likely that health-care providers did not offer this cancer prevention vaccine to the majority of study participants. Another threat may include interviewer variability. During this study, two bilingual graduate research assistants collected interview data. The graduate research assistants may have varied in the way that they interviewed participants, influencing participants’ responses. The first author tried to eliminate the threat of potential variability in interview conducted by training each graduate research assistant in interview techniques and practicing the interview with them. Finally, the research team did not ask about participants’ documentation status. Past research has shown that undocumented Latino populations have barriers to HIV primary care that impacts timeliness of HIV diagnosis and treatment as well as disease progression (when compared to documented populations).^45^ This would reduce their likelihood of learning about anal cancer and screening for anal cancer screening.

Our study’s findings highlight gaps in research. This study focused on patients’ barriers to anal cancer prevention. However, future research should explore HIV primary care providers’ practices and clinic-specific policies related to recommending the anal Pap smear among HIV-infected populations, including HIV-infected foreign-born Latino GBM. Researchers could work with health-care providers to craft linguistically and culturally appropriate messages and health education materials (including health materials communicated via various media channels) to increase awareness about the need to screen for anal cancer, focusing on ways to destigmatize this screening. Provider-led and narrated media campaigns could also begin to increase communities’ understanding and intention to screen for anal dysplasia.

Research that tests the feasibility, acceptability, and preliminary efficacy of using anal cytology self-sampling kits (paired with health education about anal cancer prevention) to increase screening for this stigmatized cancer may provide further proof of the need for these self-testing kits. Other studies should focus on how the availability of these self-testing kits affects HIV primary care practice, particularly among clinics that do not currently have the resources to test for anal dysplasia. Findings from such studies could further inform the need to make these tests more widely available.

## Conclusion

HIV-infected men are at a higher risk of developing HPV-related anal cancer. This disease can be prevented and controlled by more focused efforts to ensure that (age-eligible) men complete the HPV vaccine series and screen for anal dysplasia. It is critical to take into consideration the cultural differences reported, such as the various forms of stigma against sexual orientation, HIV, and cancer screening procedures, in order for health-care practitioners to offer more acceptable and culturally competent care. Foreign-born Latino HIV-infected GBM need information from health-care providers and the media to increase their knowledge of the HPV vaccine and anal dysplasia screening. Given the lack of screening guidelines, more work is needed to inform clinical protocols for anal dysplasia screening among HIV-infected populations. Increased health outreach and increased provider recommendations to receive the vaccine and to screen for anal cancer will provide vulnerable populations such as foreign-born Latino HIV-infected GBM with the information they need to be empowered to prevent and control HPV-related anal cancer.
